# Carotenoid Nostoxanthin Production by *Sphingomonas* sp. SG73 Isolated from Deep Sea Sediment

**DOI:** 10.3390/md19050274

**Published:** 2021-05-14

**Authors:** Hiroshi Kikukawa, Takuma Okaya, Takashi Maoka, Masayuki Miyazaki, Keita Murofushi, Takanari Kato, Yoko Hirono-Hara, Masahiro Katsumata, Shoichi Miyahara, Kiyotaka Y. Hara

**Affiliations:** 1 Department of Environmental and Life Sciences, School of Food and Nutritional Sciences, University of Shizuoka, 52-1 Yada, Suruga-ku, Shizuoka 422-8526, Japan; kikukawa@u-shizuoka-ken.ac.jp (H.K.); t.okaya007@gmail.com (T.O.); y-hirono@u-shizuoka-ken.ac.jp (Y.H.-H.); 2 Graduate Division of Nutritional and Environmental Sciences, University of Shizuoka, 52-1 Yada, Suruga-ku, Shizuoka 422-8526, Japan; keita1_murofushi@pref.shizuoka.lg.jp; 3 Research Institute for Production Development, 15 Shimogamo-Morimotocho, Sakyo-ku, Kyoto 606-0805, Japan; maoka@mbox.kyoto-inet.or.jp; 4 Institute for Extra-Cutting-Edge Science and Technology Avant-Garde Research (X-Star), Japan Agency for Marine-Earth Science and Technology (JAMSTEC), 2-15 Natsushima-cho, Yokosuka, Kanagawa 237-0061, Japan; miyazakim@jamstec.go.jp; 5 Industrial Research Institute of Shizuoka Prefecture, 2078 Makigaya, Aoi-ku, Shizuoka 421-1298, Japan;shoichi1_miyahara@pref.shizuoka.lg.jp; 6 Hagoromo Foods Corporation, 151 Shimazaki-cho, Shimizu-ku, Shizuoka 424-0823, Japan; hfc13103@hagoromofoods.co.jp (T.K.); hfc84113@hagoromofoods.co.jp (M.K.)

**Keywords:** carotenoid, nostoxanthin, *Sphingomonas*, deep sea microorganism

## Abstract

Carotenoids are used commercially for dietary supplements, cosmetics, and pharmaceuticals because of their antioxidant activity. In this study, colored microorganisms were isolated from deep sea sediment that had been collected from Suruga Bay, Shizuoka, Japan. One strain was found to be a pure yellow carotenoid producer, and the strain was identified as *Sphingomonas* sp. (Proteobacteria) by 16S rRNA gene sequence analysis; members of this genus are commonly isolated from air, the human body, and marine environments. The carotenoid was identified as nostoxanthin ((2,3,2′,3′)-β,β-carotene-2,3,2′,3′-tetrol) by mass spectrometry (MS), MS/MS, and ultraviolet-visible absorption spectroscopy (UV-Vis). Nostoxanthin is a poly-hydroxy yellow carotenoid isolated from some photosynthetic bacteria, including some species of Cyanobacteria. The strain *Sphingomonas* sp. SG73 produced highly pure nostoxanthin of approximately 97% (area%) of the total carotenoid production, and the strain was halophilic and tolerant to 1.5-fold higher salt concentration as compared with seawater. When grown in 1.8% artificial sea salt, nostoxanthin production increased by 2.5-fold as compared with production without artificial sea salt. These results indicate that *Sphingomonas* sp. SG73 is an efficient producer of nostoxanthin, and the strain is ideal for carotenoid production using marine water because of its compatibility with sea salt.

## 1. Introduction

Carotenoids are isoprenoids, and these yellow to orange-red pigments are widely distributed in nature [1]. They are predominantly synthesized by phototrophic organisms and by some non-phototrophic fungi, bacteria, and archaea [1,2]. Carotenoids are used commercially, especially astaxanthin, as a color enhancer for marine aquaculture resources such as salmon, and for dietary supplements, cosmetics, and pharmaceuticals because of its high antioxidant activity [3,4]. Industrial demand for carotenoids has recently increased, which has resulted in an increase in studies of carotenoid production, especially using marine organisms [5,6,7,8].

**Figure 1:**
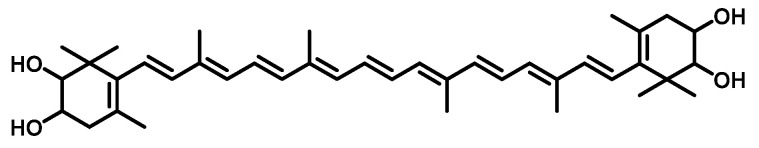
The structure of nostoxanthin.

The yellow carotenoid nostoxanthin ((2,3,2′,3′)-β,β-carotene-2,3,2′,3′-tetrol) (Figure 1) is biosynthesized from zeaxanthin by the addition of two hydroxyl groups [9]. On the basis of the chemical structure of nostoxanthin, it is expected to have a similar high antioxidative activity as that of zeaxanthin, but no study revealing the antioxidative activity of nostoxanthin has been reported. This carotenoid has been isolated from some prokaryotes including some species of Cyanobacteria: the bacteriochlorophyll-a containing bacterium *Sandarakinorhabdus limnophila*, photosynthetic bacteria, the non-marine *Brevundimonas* sp., and most *Sphingomonas* species [9,10,11,12,13,14,15,16]. However, the concentration of nostoxanthin in known nostoxanthin-producing bacteria is low [9].

In this study, we isolated a microorganism from deep sea sediment that had been collected from Suruga Bay, Shizuoka, Japan (Table 1) and we identified the chemical structure of the main carotenoid produced by the isolated microorganism. This organism showed high nostoxanthin composition in total carotenoids, and we identified the bacterial strain. Furthermore, we characterized the salt tolerance and nostoxanthin productivity by supplementation with artificial sea salt.

## 2. Results and Discussion

### 2.1. Isolation of a Yellow Microorganism from Deep Sea Sediment

**Figure 2:**
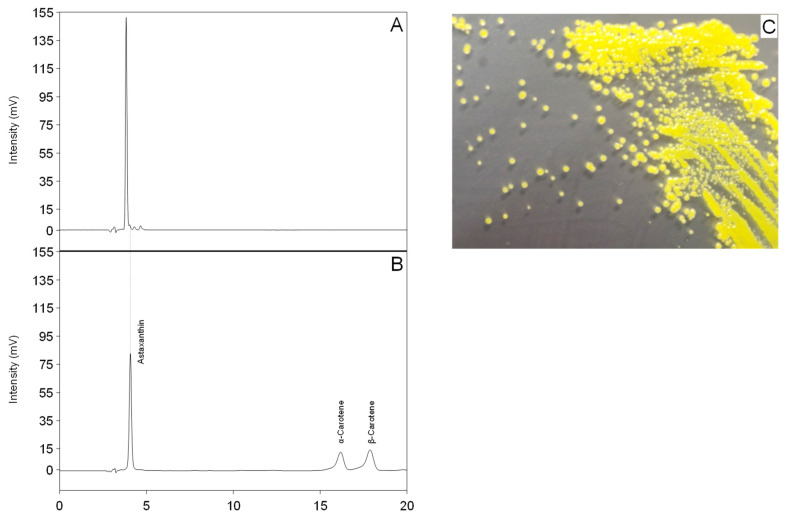
Chromatograph of carotenoids. (**A**) Carotenoids extracted from strain SG73; (**B**) authentic standards: astaxanthin, α-carotene, and β-carotene; (**C**) yellow wet colonies of strain SG73. The colonies were grown on YM agar medium at 22 °C for 3 days. The separation was performed at 35 °C, with methanol/tetrahydrofuran = 8/2 (*v*/*v*) as the mobile phase at a flow rate of 1.0 mL/min, and the detection was performed at 470 nm with an SPD-20AV detector.

Firstly, colored microorganisms were isolated from deep sea sediment. One isolate, strain SG73, from the sampling Spot 2 in Table 1, showed accumulation of highly pure carotenoid at a retention time of approximately 3.8 min and three minor peaks at a retention time of 4 to 5 min (Figure 2A). The three minor peaks were detected in the chromatography for HPLC and LC/MS (Supplemental Figure S1). The purity of the main carotenoid was 97% (area%). These carotenoids did not correspond to the peaks of authentic standards of astaxanthin, α-carotene, or β-carotene (Figure 2B). The major carotenoid from strain SG73 was expected to have higher hydrophilicity than astaxanthin because of its faster retention time.

### 2.2. Identification of the Hydrophilic Carotenoid-Producing Strain SG73

**Figure 3:**
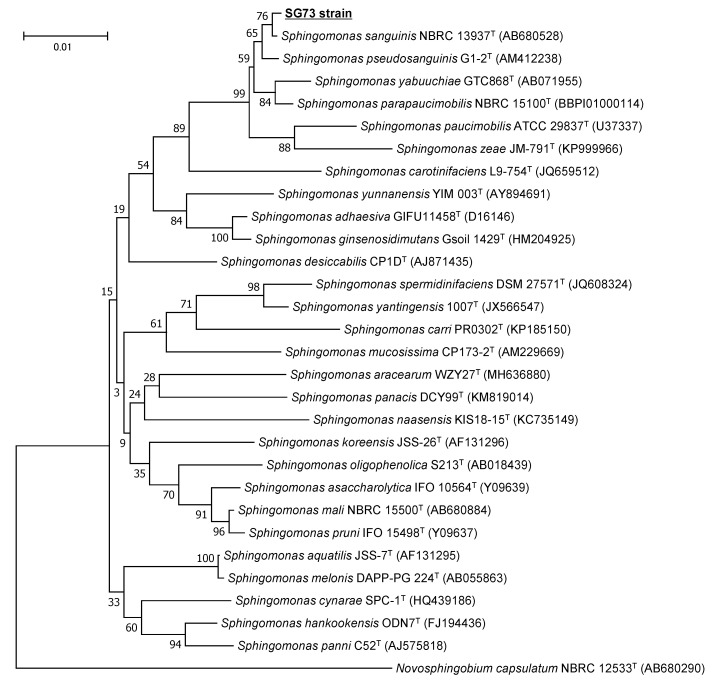
Phylogenetic tree of strain SG73 and related microorganisms created with the neighbor-joining method. Numbers indicate bootstrap values. ^T^ indicates type strain of a species.

The colony of strain SG73 exhibited a strong yellow color and a smooth surface (Figure 2C). The 16S rRNA gene sequence (1412 bp, accession no. LC618681) amplified from strain SG73 had 99.8% homology to that of *Sphingomonas sanguinis* NBRC 13937. Strain SG73 was clustered with *S. sanguinis* NBRC 13937 in the phylogenetic tree, but the bootstrap value was low (76%) (Figure 3). However, these results indicate that there may be only a small genetic distance between these strains. Therefore, strain SG73 was identified as *Sphingomonas* sp. SG73.

*Sphingomonas* is a group of Gram-negative, rod-shaped, chemoheterotrophic, aerobic bacteria. *Sphingomonas* species are known to contain glycosphingolipids [17], and are widely distributed in nature, having been isolated from many different air, land, and water habitats, including seawater and marine soils. Furthermore, *Sphingomonas* species have been reported to produce the carotenoids lycopene, β-carotene, zeaxanthin, caloxanthin, and nostoxanthin [9,13,17].

### 2.3. Identification of the Main Carotenoid Produced by Sphingomonas sp. SG73

**Figure 4:**
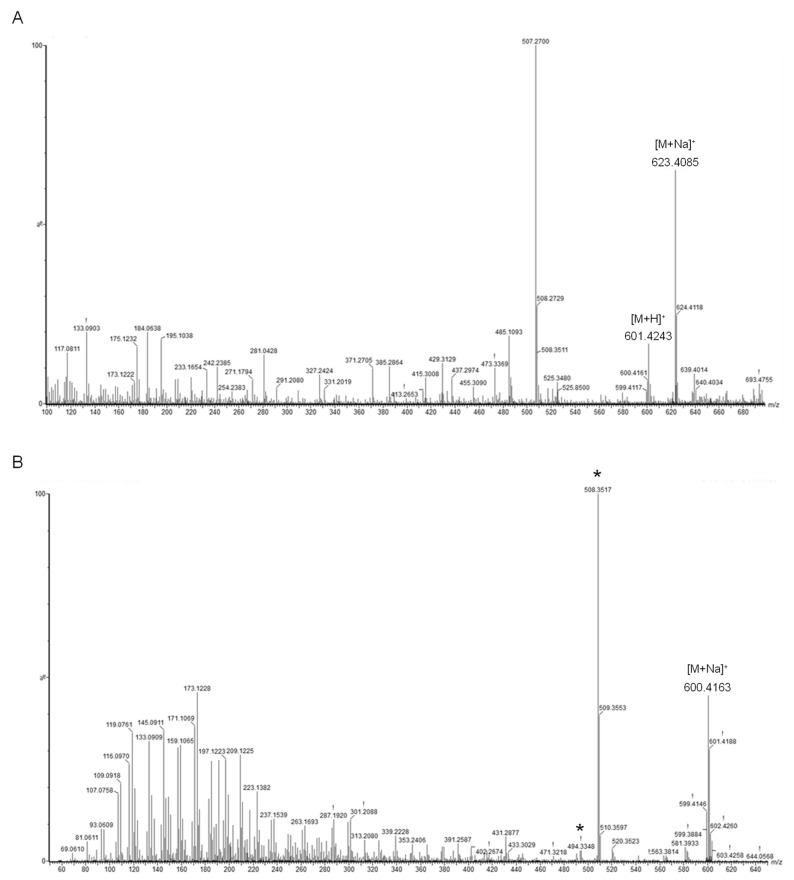

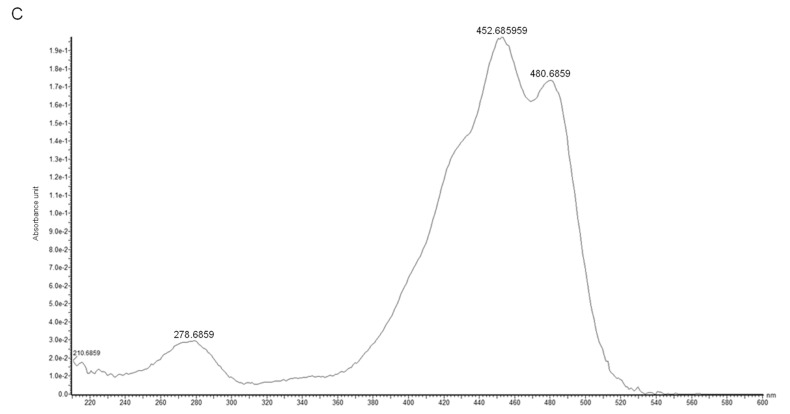
Spectra of mass spectroscopy (MS) (**A**), MS/MS (**B**), and ultraviolet visible absorption spectroscopy (UV-Vis) (**C**) of the main carotenoid from *Sphingomonas* sp. SG73. * Main product ions derived from carotenoids.

Identification of the main carotenoid produced by *Sphingomonas* sp. SG73 was conducted by high-performance liquid chromatography (HPLC) equipped with an ultraviolet-visible absorption spectrometer (UV-Vis), an electro-spray ionization (ESI) a time-of-flight (TOF) mass spectrometer (MS), and MS/MS systems. The molecular formula of this carotenoid was determined to be C_40_H_56_O_4_ by ESI MS spectra data of *m*/*z* 601.4243 [M + H]^+^ and *m*/*z* 623.4085 [M + Na]^+^ (Figure 4A). The MS/MS spectra that used precursor ion at *m*/*z* 600.41 [M^+^] showed characteristic product ion patterns of tetrahydroxy carotenoid (Figure 4B). These product ion patterns were identical with nostaoxanthin [18], and major product ions from carotenoids [19], *m*/*z* 508.3515 [M-92] generated by elimination of toluene moiety from the polyene chain and *m*/*z* 494.3283 [M-106] generated by elimination of xylene moiety from the polyene chain, were observed. The UV-Vis spectrum [λ_max_ values (427, 452, and 480 nm)] indicated the presence of β,β-carotene type chronopher (Figure 4C) [20].

**Figure 5:**
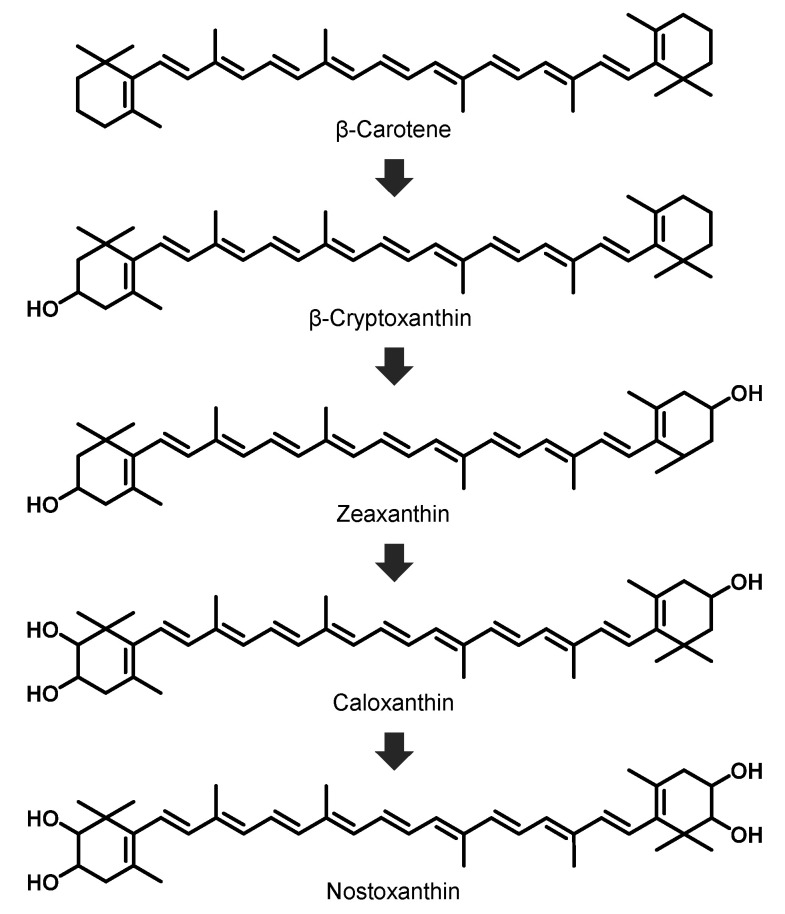
Presumed pathway of nostoxanthin biosynthesis in *Sphingomonas* sp. SG73.

These results indicate that the main carotenoid produced by *Sphingomonas* sp. SG73 is nostoxanthin ((2,3,2′,3′)-β,β-carotene-2,3,2′,3′-tetrol). Production of nostoxanthin by other *Sphingomonas* strains has been reported [9,13]; however, the major carotenoids produced by this strain were caloxanthin and zeaxanthin, and the ratio of nostoxanthin was estimated to be about 11% (area%) and less than half of the two main carotenoids. *Sphingomonas* sp. SG73, found in this study, produced nostoxanthin with high purity of approximately 97% of the total carotenoid production. A biosynthesis pathway for nostoxanthin by *Sphingomonas* species has been reported and is shown in Figure 5 [9]. Nostoxanthin is synthesized from β-carotene via four hydroxylation steps performed by two enzymes. Caloxanthin and zeaxanthin, which are the main carotenoids in *S. elodea* ATCC 31461, are precursors of nostoxanthin, and they are synthesized from β-carotene through β-cryptoxanthin.

### 2.4. Effect of Sea Salt on Growth and Nostoxanthin Production

**Figure 6:**
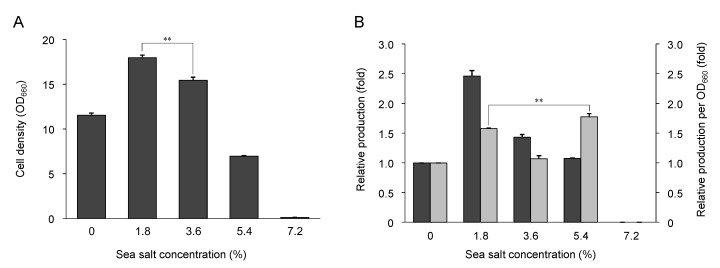
Effect of sea salt on growth and nostoxanthin production by *Sphingomonas* sp. SG73. (**A**) Cell density (OD_660_) of *Sphingomonas* sp. SG73 cultured with sea salt; (**B**) Relative production of nostoxanthin by *Sphingomonas* sp. SG73. Black bars indicate the relative production level, and gray bars indicate the relative value of production per cell density. All values are means and standard deviations for triplicate experiments. ** *p* < 0.01.

To confirm that *Sphingomonas* sp. SG73 is derived from the sea and is tolerant to sea salt, *Sphingomonas* sp. SG73 was cultured in YM liquid medium containing from 0% to 7.2% artificial sea salt. As shown in Figure 6A, the addition of 1.8% and 3.6% sea salt increased growth of *Sphingomonas* sp. SG73, and the strain grew best with 1.8% of sea salt (1.6-fold). When grown in 5.4% sea salt concentration, growth of the strain was decreased, and the strain did not grow in the medium containing 7.2% sea salt.

Nostoxanthin production in *Sphingomonas* sp. SG73 cultured with each salt concentration was analyzed by HPLC analysis. Nostoxanthin production by *Sphingomonas* sp. SG73 was highest when grown with 1.8% of sea salt and was 2.5-fold higher as compared with the amount produced without sea salt (Figure 6B). Carotenoid production per cell density was higher when cultured with 5.4% sea salt than with 1.8% sea salt. When the growth of *Sphingomonas* sp. SG73 was suppressed at higher salt concentrations, it suggested that metabolic, proteomic, and energetic flows to cell growth might be held down and their excess flows were diverted to the carotenoid biosynthetic pathway.

Growth of some *Sphingomonas* relative species with 3.6% sea salt were surveyed (data not shown). Similar to *Sphingomonas* sp. SG73, the addition of 3.6% sea salt increased growth of *Sphingomonas* sp. NBRC 101068 and NBRC 101704, which were isolated from seawater of Tokyo Bay and Pacific Ocean, respectively, whereas *Sphingomonas* sp. JCM 11416 and NBRC 13937, which were isolated from air and human blood, showed no growth. Since 3.6% sea salt is the concentration in seawater, the *Sphingomonas* strains showed favorable growth and higher nostoxanthin production under 3.6% sea salt. However, the sea salt concentration of the deep sea of Suruga Bay has been reported to be approximately 3.4% [21]. Further optimization of sea salt concentration between 1.8% and 3.6% and other metabolic and culture conditions may improve growth and nostoxanthin production of *Sphingomonas* sp. SG73.

## 3. Materials and Methods

### 3.1. Materials

The following products were used in the experiments described below: Tryptone (Thermo Fisher Scientific, Waltham, MA, USA); yeast extract and malt extract (Becton, Dickinson and Company, Franklin Lakes, NJ, USA); D-glucose (FUJIFILM Wako Pure Chemical Corporation, Osaka, Japan); and Daigo’s artificial seawater (FUJIFILM Wako Pure Chemical Corporation, Osaka, Japan). All solvents for HPLC analysis were purchased from Kanto Kagaku, Tokyo, Japan.

### 3.2. Strains and Media

The bacteria in this study were isolated from deep sea sediment of Suruga Bay, Shizuoka, Japan (Table 1). Rich yeast malt (YM) medium was used as base medium, containing 5 g/L tryptone, 3 g/L yeast extract, 3 g/L malt extract, and 10 g/L d-glucose. Deep sea microorganisms in the sediment were cultivated on YM agar medium containing 0.36% (*w*/*v*) Daigo’s artificial seawater at 22 °C for 3 to 7 days, and bacteria that grew as colored colonies were isolated.

### 3.3. Cultivation of Isolated Strains

The isolated strains were pre-cultivated in 10 mL YM liquid medium in a 50 mL baffled Erlenmeyer flask at 22 °C with agitation at 200 rpm for 24 h. Each culture was inoculated into 10 mL of fresh medium in a 50 mL baffled Erlenmeyer flask to achieve initial OD_660_ values of 0.15. Then, cells were grown at 22 °C with agitation at 200 rpm for 72 h. To evaluate the salt tolerance of isolated strains and the effect of salt on carotenoid production in the isolated strains, Daigo’s artificial seawater was supplemented into the YM liquid medium at concentrations from 0 to 7.2% (*w*/*v*); 3.6% artificial seawater is equivalent to real seawater.

### 3.4. Carotenoid Analysis

To measure the intracellular carotenoid content of the isolated strains, harvested cells were suspended in 1 mL acetone and broken using a bead shaker (Shake Master NEO, BMS, Tokyo, Japan) with 0.6 mm diameter zirconia beads. The resulting cell extract was centrifuged at 8000× *g* for 10 min at 4 °C.

Carotenoid analysis was performed using a HPLC system (Shimadzu, Kyoto, Japan) equipped with a COSMOSIL packed column Cholester (NACALAI TESQUE, Kyoto, Japan), as described previously [22]. The separation was performed at 35 °C, with methanol/tetrahydrofuran = 8/2 (*v*/*v*) as the mobile phase at a flow rate of 1.0 mL/min, and the detection was performed at 470 nm with an SPD-20AV detector (Shimadzu). Twenty microliters of each sample solution was injected.

### 3.5. Identification of Carotenoid Structure

The LC/MS analysis of carotenoids was carried out using a Waters Xevo G2S Q TOF mass spectrometer (Waters Corporation, Milford, CT, USA) equipped with an Acquity UPLC system. The ESI-TOF/MS spectra were acquired by scanning from *m*/*z* 100 to 1500 with a capillary voltage of 3.2 kV, cone voltage of 20 eV, and source temperature of 120 °C. Nitrogen was used as a nebulizing gas at a flow rate of 30 L/h. The MS/MS spectra were measured with a quadrupole-TOF MS/MS instrument with argon as a collision gas at a collision energy of 20 V. The UV-Vis absorption spectra were recorded from 200 to 600 nm using a photodiode-array detector (PDA) (Acquity UPLC PDA eλ Detector, Waters, Milford, MA, USA). An Acquity 1.7 μm BEH UPLC C18 (2.1 id X 100 mm) column (Waters Corporation, Milford, CT, USA) was used as a stationary phase and MeCN/H2O (85:15)–MeCN/MeOH (65:35) (linear gradient 0 to 15 min) as a mobile phase, at a flow rate of 0.4 mL/min for the HPLC system.

### 3.6. Genetic Analysis

The 16S rRNA gene sequence of the isolated strain was determined by TechnoSuruga Laboratory Co. Ltd., Shizuoka, Japan. The 16S rRNA gene sequence was subjected to homology searching using ENKI v3.2 (TechnoSuruga Laboratory, Shizuoka, Japan) against the Microbial Identification Database DB-BA15.0 (TechnoSuruga Laboratory, Shizuoka, Japan) and the International Nucleotide Sequence Databases (DDBJ, ENA, and GenBank). Multiple alignments were analyzed using the CLUSTAL W algorithm [23], and a phylogenetic tree was constructed using MEGA v7.0 [24] by the neighbor-joining method. The 16S rRNA gene sequences of the isolated strain and its closest relatives were identified as follows: *Sphingomonas sanguinis* NBRC 13937T (AB680528), *S. pseudosanguinis* G1-2T (AM412238), *S. yabuuchiae* GTC868T (AB071955), *S. parapaucimobilis* NBRC 15100T (BBPI01000114), *S. paucimobilis* ATCC 29837T (U37337), *S. zeae* JM-791T (KP999966), *S. carotinifaciens* L9-754T (JQ659512), *S. yunnanensis* YIM 003T (AY894691), *S. adhaesiva* GIFU11458T (D16146), *S. ginsenosidimutans* Gsoil 1429T (HM204925), *S. desiccabilis* CP1DT (AJ871435), *S. spermidinifaciens* DSM 27571T (JQ608324), *S. yantingensis* 1007T (JX566547), *S. carri* PR0302T (KP185150), *S. mucosissima* CP173-2T (AM229669), *S. aracearum* WZY27T (MH636880), *S. panacis* DCY99T (KM819014), *S. naasensis* KIS18-15T (KC735149), *S. koreensis* JSS-26T (AF131296), *S. oligophenolica* S213T (AB018439), *S. asaccharolytica* IFO 10564T (Y09639), *S. mali* NBRC 15500T (AB680884), *S. pruni* IFO 15498T (Y09637), *S. aquatilis* JSS-7T (AF131295), *S. melonis* DAPP-PG 224T (AB055863), *S. cynarae* SPC-1T (HQ439186), *S. hankookensis* ODN7T (FJ194436), *S. panni* C52T (AJ575818), and *Novosphingobium capsulatum* NBRC 12533T (AB680290).

## 4. Conclusions

In this study, a novel nostoxanthin producer *Sphingomonas* sp. SG73 was isolated from deep sea sediment that had been collected from Suruga Bay, Shizuoka, Japan. Nostoxanthin is expected to have high antioxidative activity. The strain was halophilic, and the growth and nostoxanthin production was stimulated with 1.8–3.6% sea salt. In the future, a commercial supply of nostoxanthin can be achieved by improvement of this pure nostoxanthin producer *Sphingomonas* sp. SG73.

## Figures and Tables

**Table 1:** Sampling spots in the Suruga Bay.

	Location	Water Depth
Spot 1	34°8482′ N, 138°3589′ E	376 m
Spot 2	34°8526′ N, 138°3994′ E	691 m
Spot 3	34°9149′ N, 138°6507′ E	1548 m
Spot 4	34°9152′ N, 138°6534′ E	1523 m
Spot 5	34°1872′ N, 138°4220′ E	3595 m
Spot 6	34°2736′ N, 138°4813′ E	3233 m
Spot 7	34°7847′ N, 138°6345′ E	1652 m
Spot 8	34°9867′ N, 138°6967′ E	1299 m
Spot 9	34°2409′ N, 138°4803′ E	3372 m
Spot 10	34°7835′ N, 138°6171′ E	1730 m

## Supplementary Materials

The following are available online at https://www.mdpi.com/article/10.3390/md19050274/s1, Figure S1: Chromatogram of LC/MS/MS analysis. The main peak is nostoxanthin. The small peak 1, 2, and 3 is presumed as caloxanthin, zeaxanthin, and β-cryptoxanthin, respectively.
