# Supplementary material for: Carotenoid Nostoxanthin Production by Sphingomonas sp. SG73 Isolated from Deep Sea Sediment
Source: Mar Drugs. 2021 May 14;19(5):274. doi: 10.3390/md19050274 (PMC8156329; doi:10.3390/md19050274)
Supplement: Supplementary file 1 [file marinedrugs-19-00274-s001.zip › marinedrugs-1195820-supplementary.pptx]

## Slide 1
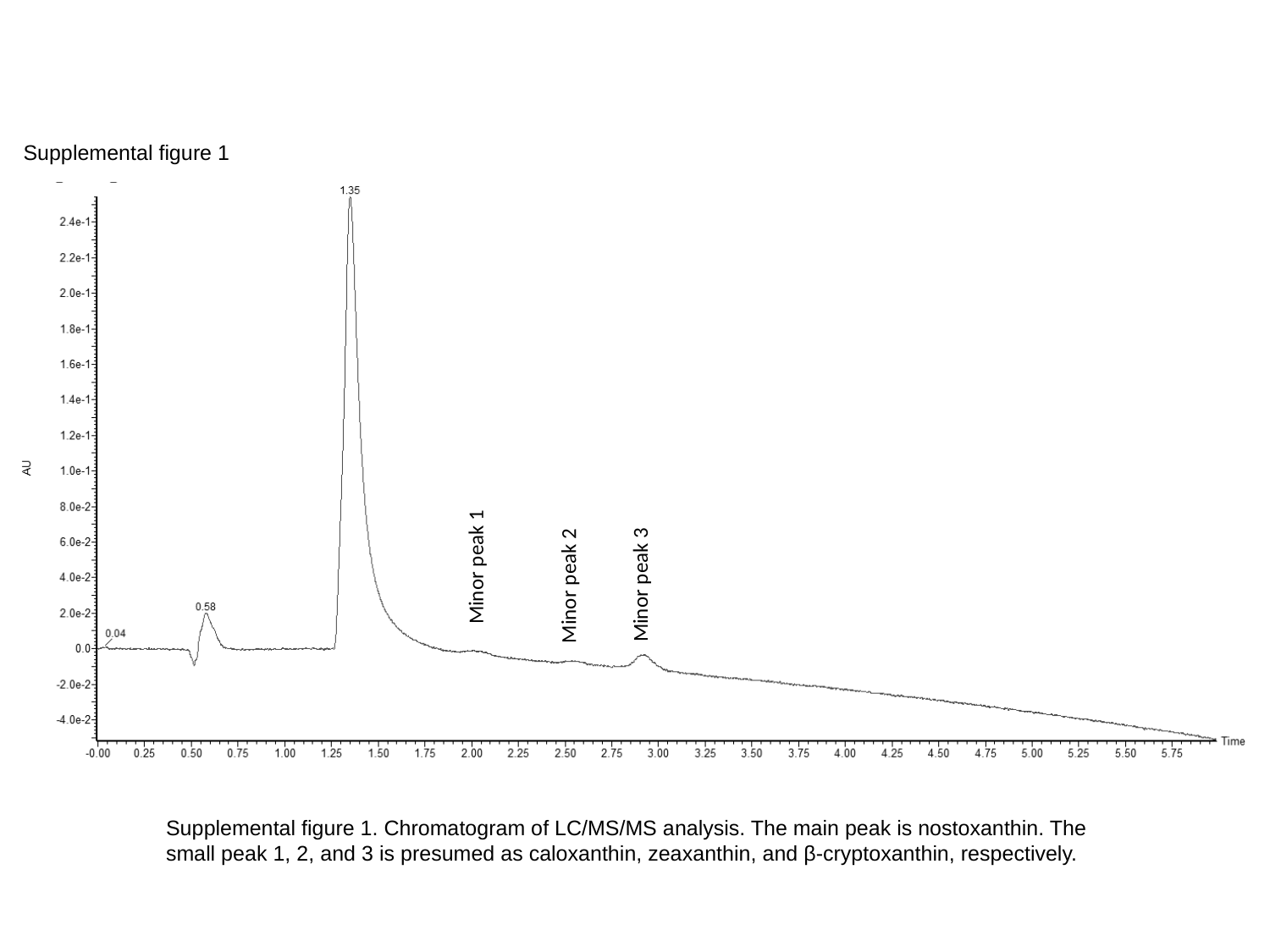

Supplemental figure 1
Minor peak 1
Minor peak 3
Minor peak 2
Supplemental figure 1. Chromatogram of LC/MS/MS analysis. The main peak is nostoxanthin. The small peak 1, 2, and 3 is presumed as caloxanthin, zeaxanthin, and β-cryptoxanthin, respectively.
